# Zwischen Regulierung und Repression: Ein Beitrag zur Einordnung gesetzlicher Maßnahmen in Bezug auf die Auslandsfinanzierung von zivilgesellschaftlichen Organisationen

**DOI:** 10.1007/s41358-021-00279-y

**Published:** 2021-08-16

**Authors:** Thomas Richter, Jessica Johansson, Silvia Rojas Castro

**Affiliations:** 1 Leibniz-Institut für Globale und Regional Studien (GIGA), Hamburg, Deutschland; 2 European Center for Constitutional and Human Rights (ECCHR), Berlin, Deutschland

**Keywords:** Schrumpfende zivilgesellschaftliche Räume, Zivilgesellschaftliche Organisationen, Auslandsfinanzierungsgesetze, Vereinigungsfreiheit, Klassifizierung gesetzlicher Maßnahmen, Shrinking civic spaces, Civil society organisations, Foreign funding laws, Freedom of association, Classification of legal measures

## Abstract

Gesetzliche Maßnahmen im Bereich der Auslandsfinanzierung von zivilgesellschaftlichen Organisationen werden inzwischen als zentraler Bestandteil von schrumpfenden zivilgesellschaftlichen Räumen angesehen. Allerdings existiert in der Literatur bisher kein Konsens über die Angemessenheit eines expliziten staatlichen Eingriffs in diesem Bereich. Der Artikel entwickelt deswegen einen konzeptionellen Rahmen, um den einschränkenden Charakter von staatlichen Eingriffen empirisch präziser als bisher bestimmen zu können. Wir plausibilisieren unser konzeptionelles Argument anhand von sechs kurzen Fallstudien zu Deutschland, Österreich, der Türkei, Ungarn, Uruguay und Venezuela. Damit tragen wir in dreifacher Art und Weise zur Debatte über schrumpfende zivilgesellschaftliche Räume bei. Wir zeigen erstens, dass gesetzliche Maßnahmen in Bezug auf die ausländische Finanzierung von Zivilgesellschaft nicht zwangsläufig als Teil eines weltweiten repressiven Trends anzusehen sind. Zweitens schlagen wir eine neue analytische Einordnung vor, die über das bisher dominante binäre Verständnis hinausgeht, welches gerade im Global Süden jeglicher Anpassung gesetzlicher Maßnahmen in diesem Bereich repressive Tendenzen unterstellt. Drittens versuchen wir eine Sicht der internationalen Menschenrechte zur Vereinigungsfreiheit mit einer auf nationalen verfassungsrechtlichen Normen fokussierten Perspektive in Verbindung zu bringen, um damit empirisch-analytisch präziser auf die in vielen Ländern existierenden rechtsnormativen Unterschiede zwischen diesen beiden Ebenen aufmerksam zu machen.

## Abstract (en)

Legal measures restricting the foreign funding of civil society organisations are seen as a central component of shrinking civic spaces. However, there is no consensus in the literature on what constitutes appropriate state intervention in this area. The article therefore develops a conceptual framework to empirically determine the restrictive character of state intervention more precisely. We make plausible our conceptual argument based on six short case studies on Germany, Austria, Turkey, Hungary, Uruguay, and Venezuela. In doing so, we contribute to the debate on shrinking civic spaces in three ways. First, we show that legal measures regarding foreign funding of civil society should not always be seen as part of a global repressive trend. Second, we propose a new classification of legal measures that goes beyond the hitherto dominant binary understanding that, especially regarding the Global South, assumes repressive tendencies to any adaptation of legal measures. Thirdly, we attempt to link a view of international human rights on the freedom of association with a perspective focused on national constitutional norms, to draw attention to the existing legal normative differences between these two levels in many countries around the world.

## Einleitung

Schrumpfende zivilgesellschaftliche Räume,^1^ sogenannte *shrinking civic spaces*, haben in den letzten Jahren massiv an Bedeutung gewonnen. Nicht zuletzt seit dem Beginn der COVID-19-Pandemie im Frühjahr 2020 haben Regierungen in der ganzen Welt damit begonnen, zivilgesellschaftliche Aktivitäten zusätzlich einzuschränken (Bethke und Wolff 2020; Brown et al. 2020). Diese Entwicklungen haben schon seit einigen Jahren bei Aktivist:innen aber auch bei internationalen Gebern und in der Wissenschaft für eine besorgte Aufmerksamkeit gesorgt (vgl. beispielsweise Borgh und Terwindt 2012; Carothers und Brechenmacher 2014; Dupuy et al. 2016; Unmüßig 2016; Anheier et al. 2018). Obwohl historisch gesprochen staatliche Restriktion zivilgesellschaftlichen Handelns keine neuartige Entwicklung darstellt (Richter 2018), scheint insbesondere der seit Beginn der 2000er-Jahre zu beobachtende Anstieg formaler Barrieren für die ausländische Finanzierung zivilgesellschaftlicher Organisationen (ZOs) eine neue Form im Portfolie staatlicher Regulierung darzustellen (Sikkink 2018, S. 175). Etwa ein Drittel der seit der Jahrtausendwende zusätzlich implementierten weltweiten staatlichen Maßnahmen fällt in diese Kategorie (Rutzen 2015a, S. 5). Inzwischen ist eine wie auch immer geartete spezifische Regulierung der Auslandsfinanzierung von ZOs in allen Weltregionen und sowohl in Autokratien als auch in Demokratien anzutreffen (Carothers und Brechenmacher 2014; Baldus 2015; Dupuy et al. 2016; Buyse 2018).

Über die Angemessenheit des expliziten staatlichen Eingriffs in diesem Bereich existiert in der Literatur kein Konsens (vgl. dazu beispielsweise Kiai 2013; Rutzen 2015a; Poppe und Wolff 2016, 2017; Buyse 2018). Im Kern wird darüber gestritten, ob gesetzliche Maßnahmen mit Bezug auf eine Auslandsfinanzierung von Zivilgesellschaft das Recht auf Vereinigungsfreiheit einschränken. Und falls ja, welche angemessenen Gründe für eine solche Einschränkung existieren, damit staatliche Regulierung nicht zur Repression wird. Maina Kiai, der ehemalige VN-Sonderberichterstatter für das Recht auf Versammlungs- und Vereinigungsfreiheit, betrachtet den uneingeschränkten Zugang zu allen in- und ausländischen Ressourcen als eine notwendige Voraussetzung für die Ausübung der Vereinigungsfreiheit. „Hence, undue restrictions on resources available to associations impact the enjoyment of the right to freedom of association and also undermine civil, cultural, economic, political and social rights as a whole“ (Kiai 2013, S. 5). Annika Poppe und Jonas Wolff (Wolff und Poppe 2015; Poppe und Wolff 2017) hingegen argumentieren, dass es kein *a priori* Recht der Zivilgesellschaft auf externe finanzielle Unterstützung gäbe. Argumente, die für eine uneingeschränkte ausländische Finanzierung von Zivilgesellschaft sprächen, so Poppe und Wolff, beruhten auf normativ umstrittenen liberalen Annahmen über die bestehende Weltordnung. „[G]overnments and non-state actors that want to increase the political space of civil society […] should accept that countries do, and legitimately can, impose specific restrictions on foreign funding“ (Wolff und Poppe 2015, S. 33). Eine Restriktion von Auslandsfinanzierung sei insbesondere dann angemessen, wenn es darum ginge, politische Einmischung von außerhalb zu unterbinden.

Ausgehend von dieser Debatte möchten wir in diesem Beitrag genauer diskutieren ob und unter welchen Bedingungen eine gesetzliche Maßnahme zur Auslandsfinanzierung von zivilgesellschaftlichen Organisationen eine angemessene staatliche Regulierung bleibt und wann sie gegebenenfalls als Repression einzuordnen ist. Um diese Fragestellung beantworten zu können, schlagen wir einen konzeptionellen Rahmen vor, der sowohl verfassungsrechtliche als auch internationale Rechtsnormen berücksichtigt, um für eine politikwissenschaftliche Leserschaft den einschränkenden Charakter von durch Recht kodifizierten staatlichen Eingriffen empirisch präziser als bisher bestimmen zu können. Konkret entwickeln wir einen Kriterienkatalog, der in vereinfachter Form hilft zu unterscheiden, ob es sich bei den jeweiligen in Gesetzesform formulierten staatlichen Maßnahmen um eine angemessene Regulierung, um eine gerechtfertigte Einschränkung oder eben möglicherweise um Repression handelt.

Damit versuchen wir in dreifacher Art und Weise zu den bisherigen Debatten über schrumpfende zivilgesellschaftliche Räume beizutragen. Wir zeigen erstens, dass gesetzliche Maßnahmen in Bezug auf die ausländische Finanzierung von Zivilgesellschaft nicht zwangsläufig als Teil eines weltweiten repressiven Trends anzusehen sind. Zweitens schlagen wir eine diesem Einwand gerecht werdende neue empirisch-analytische Einordnung vor, die über das bisher dominante Verständnis hinausgeht, welches gerade im Global Süden jeglicher Anpassung gesetzlicher Maßnahmen in diesem Bereich repressive Tendenzen unterstellt. Drittens versuchen wir, die internationale Menschenrechtsperspektive zur Vereinigungsfreiheit mit einer Perspektive in Verbindung zu bringen, die auf nationale verfassungsrechtliche Normen fokussiert, um damit besser auf die in vielen Ländern existierenden Widersprüche zwischen diesen beiden Ebenen aufmerksam zu machen.

Unser Beitrag ist folgendermaßen aufgebaut. Im sich anschließenden zweiten Abschnitt diskutieren wir drei Defizite in der Literatur an der Schnittstelle von schrumpfenden zivilgesellschaftlichen Räumen und ausländischer Finanzierung von ZOs. Darauf aufbauend schlagen wir im dritten Abschnitt einen konzeptionellen Rahmen vor, der eine präzisere Einordnung gesetzlicher Maßnahmen in diesem Bereich über eine größere Anzahl von Ländern hinweg ermöglicht. Um den empirischen Mehrwert unseres Vorschlags zu demonstrieren, führen wir im vierten Teil des Artikels eine Plausibilitätsprüfung durch. Dabei diskutieren wir den Charakter gesetzlicher Maßnahmen in Bezug auf die Auslandsfinanzierung von ZOs in sechs unterschiedlichen Staaten (Deutschland, Österreich, Türkei, Ungarn, Uruguay, und Venezuela) und ordnen die jeweilige nationale Gesetzgebung anhand des von uns vorgeschlagenen heuristischen Rahmens auf einer Skala von ermöglichender Regulierung, gerechtfertigter Einschränkung und ungerechtfertigter Einschränkung ein. Allein die letzte Kategorie bezeichnen wir als Repression. In unseren abschließenden Schlussfolgerungen unterstreichen wir das Potenzial unseres Beitrags für die zukünftige Forschung zu schrumpfenden zivilgesellschaftlichen Räumen und verweisen auf eine Reihe von zukünftigen Forschungsdesiderata.

## Literaturüberblick und Forschungsdefizite

Die Einhaltung der Vereinigungs‑, Versammlungs- und Redefreiheit durch den Staat gilt als Voraussetzung für die Existenz einer lebendigen Zivilgesellschaft. Experten haben daher davor gewarnt, dass eine zusätzliche Regulierung der ausländischen Finanzierung von ZOs, Verpflichtungen im Rahmen des Völkerrechts und der internationalen Menschenrechten verletzen könnte (Kiai 2013, 2016; Buyse 2018; American Bar Association 2015). Nach dieser Auffassung ist der uneingeschränkte Zugang zu Finanzmitteln eine notwendige Bedingung für die Ausübung dieser Grundrechte. Eine wie auch immer ausgestaltete Einschränkung der Finanzierung aus nationalen oder internationalen Quellen könnte direkt in die Vereinigungsfreiheit eingreifen (Kiai 2013) und müsste daher eine Reihe von Bedingungen erfüllen (Buyse 2018, S. 980).

Aufgrund des strukturellen Mangels an lokalen Finanzmitteln sind insbesondere weite Teile der Zivilgesellschaft im Globalen Süden faktisch abhängig von ausländischer Unterstützung. Dies gilt insbesondere für Organisationen, die sich in politisierten Bereichen wie Menschenrechte, Demokratie, Wahlen, Transparenz, Antikorruption und gute Regierungsführung engagieren. Eine Regulierung in Bezug auf ausländische Finanzierung kann sich darüber hinaus aber auch auf andere staatliche Funktionen auswirken. Sind doch in Bereichen wie Bildung, Gesundheit oder lokale Entwicklung zivilgesellschaftliche Organisationen aktiv, die im Auftrag oder an Stelle des Staates eine Grundversorgung bereitstellen oder andere essenzielle Dienstleistungen erbringen und dafür vorwiegend auf ausländische Finanzmittel angewiesen sind (Dupuy et al. 2015; Amnesty International 2016; Dupuy und Prakash 2018).

Der Großteil der bisherigen Literatur neigt dazu, neue gesetzliche Maßnahmen zur Auslandsfinanzierung als Teil des breiteren Phänomens schrumpfender zivilgesellschaftlicher Räume anzusehen (so z. B. Carothers 2006; Gershman und Allen 2006; Borgh und Terwindt 2012; Carothers und Brechenmacher 2014; Mendelson 2015; Rutzen 2015a, b; Buyse 2018). Darin wird die Regulierung der Auslandsfinanzierung als eine staatliche Strategie interpretiert, die der aktiven und präventiven Unterdrückung von zum herrschenden Regime oder der gewählten Regierung alternativen politischen Positionen dient. Insbesondere soll damit der Einfluss oppositioneller politischer Kräfte eingedämmt werden. Deren Aufstieg ist als Teil externer Demokratieförderung in vielen Fällen maßgeblich durch finanzielle Unterstützung aus dem Ausland ermöglich worden (Christensen und Weinstein 2013; Kiai 2013; Carothers und Brechenmacher 2014; Rutzen 2015a).

Inwieweit jedoch gesetzliche Maßnahmen zur Regulierung einer ausländischen Finanzierung von zivilgesellschaftlichen Tätigkeiten als Teil eines allgemeineren Trends von schrumpfenden Räumen anzusehen ist, bleibt umstritten. Selbst wenn Regierungen neue gesetzliche Maßnahmen in Bezug auf ausländische Finanzierung eingeführt haben, sind nicht in jedem Fall dazu zusätzliche Einschränkungen von zivilgesellschaftlichem Handeln implementiert worden (Carothers und Brechenmacher 2014, S. 16). Daher haben einige Autor:innen vorgeschlagen, dass der Bereich der Auslandsfinanzierungsregulierung nicht zwangsläufig als ein Teil von restriktiverem staatlichem Handeln gegenüber Zivilgesellschaft anzusehen ist. Man solle sich daher nicht nur darauf konzentrieren, ob neue staatliche Maßnahmen erlassen werden, sondern vielmehr genauer prüfen von welcher Art die dadurch entstehenden Einschränkungen tatsächlich sind (Wolff und Poppe 2015).

Ein sich daran anschließendes Defizit in der existierenden Literatur ergibt sich in Bezug auf die empirische Messung von regulativen Einschränkungen im Bereich der Auslandsfinanzierung von ZOs. Die bisher an einer Hand abzuzählenden empirisch-vergleichenden Studien zu diesem Thema verwenden alle eine binäre Kategorisierung und messen damit entweder das Vorhandensein oder das Fehlen von neuen gesetzlichen Maßnahmen in diesem Bereich. Dabei nehmen diese Arbeiten an, dass mit einem neuen Gesetz Einschränkungen einhergehen (vgl. dazu insbesondere Dupuy et al. 2016; Reddy 2018; aber auch Wolff und Poppe 2015). Ob dies aber tatsächlich der Fall ist oder welche Einschränkungsart sich durch eine spezifische staatliche Maßnahme tatsächlich ergibt, lässt sich mit dieser Vorgehensweise nicht feststellen. Zum Beispiel muss sich eine staatlich vorgeschrieben Berichtspflicht in Bezug auf ausländische Finanzierung nicht notwendigerweise negativ auf die Ausübung der Vereinigungsfreiheit auswirken. Eine neue Gesetzgebung könnte beispielsweise Gemeinwohl orientiert formuliert sein, um die finanziellen Transaktionen eines bisweilen chaotisch operierenden Sektors transparenter zu gestalten. Ein vollständiges oder teilweises Verbot von ausländischer Finanzierung hingegen würde mit hoher Wahrscheinlichkeit in die Tätigkeitsschwerpunkte und womöglich sogar in die Existenzgrundlage von ZOs eingreifen und damit die Vereinigungsfreiheit maßgeblich beschränken. Kurz gesagt, das Regulierungsdesign und das Ausmaß, in dem gesetzliche Maßnahmen in das Grundrecht auf Vereinigungsfreiheit eingreifen, scheint von Bedeutung für die empirische Einordnung zu sein, ob es sich dabei nur um einen regulativen oder bereits um einen repressiven Eingriff handelt. Über diese Unterscheidung wird in der bisherigen Literatur zur ausländischen Finanzierung von ZOs bisher nicht ausreichend reflektiert.

Ein letzter defizitärer Bereich in der aktuellen Literatur betrifft die Auswahl der normativen Bezugspunkte, um zwischen einem regulativen und einem restriktiven Charakter von staatlichen Maßnahmen unterscheiden zu können.^2^ Die Position, dass es sich bei der Mehrheit der neuen Auslandsfinanzierungsgesetze um nicht zu rechtfertigende Eingriffe in die Vereinigungsfreiheit, d. h. möglicherweise sogar um staatliche Repression handeln könnte, wurde dahingehen kritisiert, dass sie deren möglichen umstrittenen Charakter im internationalen System der Staaten nicht ausreichend berücksichtigt. Beispielsweise wird oft übersehen, welche guten Gründe Regierungen haben, um eine ausländische Finanzierung der Zivilgesellschaft einzuschränken (Wolff und Poppe 2015, 2017). In diesem Zusammenhang wurde Kritik an einer ausschließlich völkerrechtsbasierten Interpretation geübt, die die neuen Gesetzgebungen zur ausländischen Finanzierung als unumstößliches Indiz für staatliche Repression unter Verletzung internationaler Menschenrechtsverträge charakterisiert. So argumentieren z. B. Poppe und Wolff, „the political controversy over civil society support is characterized by norm contestation, and this contestation reveals competing perceptions of in/justice“ (2017, S. 469). Bereits Breen (2015) stellt gleichlautend fest, dass es im Bereich der Auslandsfinanzierung zivilgesellschaftlicher Aktivitäten einen grundsätzlichen Widerspruch zwischen dem Prinzip der Autonomie von Zivilgesellschaft basierend auf der Vereinigungsfreiheit einerseits und dem Anspruch auf nationale Souveränität andererseits gäbe. Da die uneingeschränkte ausländische Finanzierung von ZOs im Prinzip durch internationale Menschenrechtsnormen abgedeckt ist, kommen zahlreiche Analysen zu dem Schluss, dass viele der neueren gesetzlichen Maßnahmen zur ausländischen Finanzierung von Zivilgesellschaft gegen internationale Völkerrechtsprinzipien verstoßen würden (Kiai 2013, 2016; European Commission for Democracy through Law (Venice Commission) und OSCE Office for Democratic Institutions and Human Rights (OSCE/ODIHR) 2018; American Bar Association (2015)). Allerdings hat keine dieser und der wenigen empirisch-vergleichenden Arbeiten (z. B. Dupuy et al. 2016; Reddy 2018) die internationale Sicht mit einer Analyse der nationalstaatlichen Ebene verbunden. D. h. es ist bisher nicht ausreichend untersucht, ob sich gesetzliche Maßnahmen, die potenziell im Konflikt mit internationalen Menschenrechtsprinzipen stehen, möglicherweise mit Bezug auf Rechtsnormen aus dem jeweils spezifischen nationalen Verfassungsrecht rechtfertigen lassen.

## Ein politikwissenschaftlicher Einordnungsversuch gesetzlicher Maßnahmen zur Auslandsfinanzierung von ZOs

Im Hinblick auf die skizzierten Defizite in der bisherigen Literatur schlägt dieser Artikel einen konzeptionellen Rahmen vor, der eine präzisere Einordnung von gesetzlichen Maßnahmen zwischen staatlicher Regulierung und Repression ermöglicht. Unser Ziel ist es, einen konzeptionellen Weg zu finden, um Einschränkung(en) der Vereinigungsfreiheit im Bereich der Auslandsfinanzierung über eine möglichst große Anzahl von Ländern hinweg valider als bisher messen und damit miteinander vergleichen zu können. Aus forschungspragmatischen Gründen folgen wir dabei der Literatur zu etablierten Demokratien und beschränken uns auf kodifiziertes nationales Recht in Form von offiziellen staatlichen Dokumenten mit Gesetzescharakter (vergleiche dafür z. B. Bolleyer 2018) und lassen öffentliche Diskurse, rechtswidriges Verhalten durch staatliche Akteure, die informelle Einflussnahme auf die Judikative oder das Problem von (bewusst) vage gehaltenen gesetzlichen Bestimmungen unberücksichtigt. Die zuletzt genannten Aspekte haben in den letzten Jahren zu Recht eine wichtige Rolle in den Debatten um schrumpfende zivilgesellschaftliche Räume gespielt (vergleiche dafür z. B. Brechenmacher 2017; Unmüßig 2016). Für die im Entstehen begriffene empirisch-vergleichende Analyse des Phänomens erscheint es uns allerdings geeigneter, sich auf eine Auswertung der für viele Länder bereits gut dokumentierten rechtlichen Dokumente zu diesem Problembereich zu konzentrieren. Eine vergleichende Untersuchung der vielfältigen Formen der (Nicht)Anwendung und unterschiedlichen Auslegung rechtlicher Maßnahmen wäre selbst für die in diesem Artikel diskutierten sechs Fällen nicht umzusetzen gewesen. Allerdings hat beispielsweise die vergleichende politikwissenschaftliche Forschung zu Verfassungen gezeigt, dass kodifizierte Rechtsquellen selbst in Autokratien keineswegs komplett irrelevant sind. Denn insbesondere verfassungsrechtliche Texte können selbst in ihrer frühesten Form als ein normatives Drehbuch (normativ script) interpretiert werden, welches einerseits als Ergebnis einer Interaktion von sozialen Akteuren entstanden ist und auf welches andererseits in zukünftigen sozialen Interaktionen Bezug genommen wird (Elkins 2010).

Als gesetzliche Maßnahmen definieren wir alle Regeln, die in Gesetzesform schriftlich und entsprechend dem jeweils länderüblichen Rechtssystem durch staatliche Organisationen beschlossen und veröffentlicht worden sind. Wir beginnen unseren Vorschlag mit der Annahme, dass eine neue gesetzliche Maßnahme, die sich mit der Auslandsfinanzierung von ZOs beschäftigt, nicht von Natur aus restriktiv oder repressiv zu sein hat. Im Gegenteil, Staaten sollten sich grundsätzlich darum bemühen, neu entstehende Übergänge und Grenzen zwischen Staat, Wirtschaft und Zivilgesellschaft auszugestalten und damit zu verrechtlichen (so z. B. Anheier 2017, S. 4). Tatsächlich verdeutlichen gerade die Vielfalt und die stetig weiterwachsende Zahl an ZOs sowie ihre weltweit zunehmende soziale und politische Bedeutung die Notwendigkeit einer Neuformulierung und gegebenenfalls Modernisierung von gesetzlichen Maßnahmen mit Bezug auf den zivilgesellschaftlichen Sektor. D. h. gesetzliche Maßnahmen, die die Beziehung zwischen Staat und Zivilgesellschaft durch Regeln strukturieren sind so lange angemessen, wie sie das Grundrecht auf Vereinigungsfreiheit nicht verletzen und die dadurch definierte Autonomie von ZOs respektiert (Unmüßig 2016, S. 2).

Zunächst kann eine Regel bzw. eine regelorientierte Formulierung in Gesetzestexten grundsätzlich dahingehend beurteilt werden, ob sie bestehende (nationale oder internationale) Rechtsnormen einschränkt oder nicht (Alexy 2002, S. 217; Bolleyer 2018, S. 33; Buyse 2018, S. 980).^3^ Nicht einschränkende Regeln definieren den Rahmen, der zur Verwirklichung eines bestimmten Grundrechts zur Verfügung stehen soll, indem sie die Konkretisierung und Umsetzung einer Verfassungsnorm oder einer Verpflichtung gegenüber einer Völkerrechtsnorm darlegen. Für den Zweck unseres Einordnungsvorschlags definieren wir dies als *ermöglichende Regulierung*. Im Gegensatz dazu legen einschränkende Regeln Bedingungen fest, die die Ausübung einer Rechtsnorm begrenzen. Wir bezeichnen diese daher als *einschränkende Regulierung*. Schließlich kann innerhalb der zuletzt genannten Kategorie eine Abstufung zwischen *gerechtfertigt* und *ungerechtfertigt *vorgenommen werden. Wir verwenden hier einen Rechtfertigungsbegriff, so wie er in weiten Teilen der Rechtswissenschaften Verwendung findet. Es geht dabei darum zu beurteilen, ob sich eine *einschränkende Regulierung* an eine vordefinierte, oft höherrangige verfassungsrechtliche oder völkerrechtliche Rechtsnorm hält oder nicht.^4^ Die Verwendung eines rechtswissenschaftlichen Rechtfertigungsbegriffs ist für unseren Zweck aus einer Reihe von Gründen hilfreich.^5^ Nahe liegend ist dies zunächst vor allem deswegen, weil wir uns wie oben diskutiert im Regelfall auf eine Analyse nationaler rechtlicher Quellen konzentrieren, um eine präzisere Einordnung von gesetzlichen Maßnahmen vornehmen zu können. Zweitens ist die Ausübung der Vereinigungsfreiheit als Grundrecht nicht absolut. Einschränkungen von Grundrechten sind zulässig, wenn sie beispielsweise mit anderen gleichrangigen Rechten oder staatlichen Prinzipien kollidieren. Drittens kann die von uns gewählte Herangehensweise helfen eine bessere Bewertung der konkurrierenden normativen Annahmen abzugeben, die einem Teil der oben zusammengefassten Debatten über ausländische Finanzierung und schrumpfenden zivilgesellschaftlichen Räumen zugrunde liegt. Mit einer solchen Vorgehensweise wird schließlich auch für Politikwissenschaftler:innen erkennbar, dass Staaten einerseits angemessene Gründe vorbringen können, um die ausländische Finanzierung von ZOs einzuschränken. Andererseits dürfen diese Gründe nicht willkürlich gewählt sein und sollten daher entweder nationalen oder internationalen Standards – in diesem Fall Rechtsnormen – unterliegen. Schließlich werden mit einer Gegenüberstellung von Rechtfertigungen für eine mögliche Einschränkung der Vereinigungsfreiheit auch die empirisch existierenden Unterschiede und Widersprüche zwischen internationalem Völkerrecht und jeweils nationalem Verfassungsrecht deutlicher. Daraus können sich daher interessante zusätzliche Impulse ergeben, die es wert sind, zukünftig ausführlicher untersucht zu werden.

Auf Basis dieser Überlegungen schlagen wir vor, gesetzliche Maßnahmen zur Auslandsfinanzierung von ZOs, anhand der drei folgenden Kategorien zu unterscheiden:

1. *Ermöglichende Regulierung* = eine staatliche Regel, welche die Umsetzung und Konkretisierung von Auslandsfinanzierung für ZOs festschreibt.
2. *Gerechtfertigt einschränkende Regulierung* = eine staatliche Regel, die eine auf Rechtsnormen begründete Einschränkungen einer ausländischen Finanzierung für ZOs festschreibt.
3. *Ungerechtfertigt einschränkende Regulierung* = eine staatliche Regel, die eine Einschränkung der ausländischen Finanzierung von ZOs festschreiben, die sich nicht mit Bezug auf Rechtsnormen begründen lässt. Diese Maßnahmen bezeichnen wir zudem als Repression.

Beispielsweise würde ein gesetzliches Dokument, welches ausschließlich darlegt, dass ZOs ausländische Mittel verwenden können und die dafür einzuhaltenden Regeln beschreibt, als eine *ermöglichende Regulierung* eingeordnet werden. Ein in einem Dokument formulierte *einschränkende Regulierung* hingegen würde Bedingungen für den Empfang von finanziellen Ressourcen aus dem Ausland definieren, wie z. B., dass diese überhaupt nicht angenommen werden dürfen, über bestimmte Banken oder staatliche Stellen geleitet werden müssen, oder nur zugunsten bestimmter Themenbereiche ins Land fließen dürfen. D. h. sollte sich ein Hinweis darauf finden, dass der Zufluss von ausländischen Finanzmitteln in irgendeiner Form begrenzt wird, ordnen wir die gesetzliche Maßnahme als *einschränkende Regulierung* ein. Für die Entscheidung, ob eine als *einschränkende Regulierung* identifizierte Regel entweder als *gerechtfertigt* oder *ungerechtfertigt* eingeordnet werden kann, bieten sich grundsätzlich zwei Rechtsquellen an; zum einen das nationale Verfassungsrecht und zum anderen das internationale Völkerrecht, hier insbesondere die internationalen Menschenrechte. Im Folgenden diskutieren wir, anhand welcher Kriterien eine empirische Einordnung erfolgen kann.

### Einschränkungen auf Grundlage der internationalen Menschenrechte

Die Vereinigungsfreiheit ist als internationale Menschenrechtsnorm in Artikel 22 des Internationalen Pakts über bürgerliche und politische Rechte (engl. International Covenant on Civil and Political Rights = ICCPR) formuliert (UN General Assembly 1966). Hier sind drei Bedingungen benannt, die es ermöglichen dieses Grundrecht einzuschränken. Einschränkungen müssen (i) rechtmäßig sein, (ii) einem legitimen Ziel entsprechen und (iii) im Rahmen der demokratischen Ordnung als notwendig angesehen werden. Sollte auch nur eines dieser Kriterien nicht erfüllt sein, ist eine in der Gesetzgebung formulierte Einschränkung als ungerechtfertigt einzuordnen (vgl. dazu auch Buyse 2018, S. 980).

Unter der Bedingung von Rechtmäßigkeit (i) müssen Einschränkungen gesetzlich formuliert, zugänglich und verständlich sein, damit betroffene Akteure ihr Verhalten entsprechend anpassen können (European Commission for Democracy through Law, Venice Commission und OSCE Office for Democratic Institutions and Human Rights, OSCE/ODIHR 2014). Die Bedingung eines legitimen Ziels (ii) legt darüber hinaus fest, dass Einschränkungen nur dann gerechtfertigt sind, wenn sie zusätzlich die in Artikel 22 ICCPR benannten Ziele verfolgen:^6^ nationale oder öffentliche Sicherheit, öffentlichen Ordnung (ordre public), Schutz der öffentlichen Gesundheit, der öffentlichen Sittlichkeit, oder der Rechte und Freiheiten Dritter. Wenn also eine in einem Gesetz formulierte Einschränkung keinen oder einen Grund benennt, der nicht in Artikel 22 ICCPR aufgeführt ist, dann ordnen wir diese Regel der Kategorie *ungerechtfertigt einschränkende Regulierung* zu. Sollte allerdings ein legitimes Ziel der Einschränkung benannt sein, muss schließlich geprüft werden, ob diese Einschränkung als notwendig für die demokratische Ordnung (iii) angesehen werden kann. Diese Einschätzung beruht rechtswissenschaftlich ausgedrückt auf einer Verhältnismäßigkeitsprüfung im engeren Sinne. Kurz gesprochen, es muss entscheiden werden, ob die in einer Gesetzgebung formulierte Beschränkung, die am wenigsten einschränkende Möglichkeit benennt, um das vorab im Gesetzestext benannte legitime Ziel der Einschränkung zu erreichen (European Commission for Democracy through Law, Venice Commission und OSCE Office for Democratic Institutions and Human Rights, OSCE/ODIHR 2014; Buyse 2018, S. 981). Für die konkrete empirische Einordnung einer Einschränkung schlagen wir für diesen letzten Schritt vor, sich an den vom Europarat praktizierten Standards bzw. Einschätzungen, die insbesondere durch die Venedig-Kommission und den europäischen Menschengerichtshof geprägt sind, zu orientieren. Hier existiert ein Korpus von Gutachten und Entscheidungen, die bereits detaillierte Hinweise darauf geben, ob eine mögliche Einschränkung tatsächlich die Bedingung der Notwendigkeit in einer demokratischen Ordnung erfüllt oder nicht.

### Einschränkungen auf Grundlage des nationalen Verfassungsrechts

Die Einordnung einer Einschränkung auf Basis von nationalen verfassungsrechtlichen Rechtsnormen zielt gleichermaßen darauf hin, zu bestimmen, ob neue gesetzliche Maßnahmen ein Grundrecht auf Vereinigungsfreiheit einschränken. Und wenn ja, ob diese Einschränkung (nicht) zu rechtfertigen ist. Leider gibt es für ein solches Vorgehen keine global einheitliche normative Rechtsquelle. Eine Einordnung ist daher im Rahmen des jeweils spezifischen nationalen Verfassungsrechts zu erfolgen. Allerdings ist es auch hier möglich, sich an den Grundelementen der Verhältnismäßigkeitsprüfung zu orientieren (Alexy 2002; Sweet und Mathews 2008; Barak 2012), die zunehmend auch für international vergleichende Verfassungsanalysen herangezogen werden. Ganz ähnlich wie für den Bereich der internationalen Menschenrechte gilt es die folgenden vier Prinzipen für eine Einordnung heranzuziehen: (I) Rechtmäßigkeit, (II) Legitimität, (III) Angemessenheit und (IV) Notwendigkeit.

In Bezug auf Rechtmäßigkeit (I) ist also zunächst erneut zu prüfen, ob neue gesetzliche Maßnahmen den rechtlichen Gepflogenheiten des jeweiligen Landes entsprechend erlassen wurden. Bei der weitergehenden Einschätzung in Bezug auf Legitimität (II) geht es darum herauszufinden, ob der im jeweiligen Gesetz benannte Grund für die spezifische Einschränkung so auch in der jeweiligen Verfassung als legitimer Grund für eine mögliche Einschränkung einer Grundrechtsnorm benannt ist (Barak 2012, S. 245 ff.; Jackson 2014, S. 3111–3113).^7^ Dies kann nur nach einer Analyse der jeweils relevanten Gesetzestexte, der Verfassung und gegebenenfalls weiterer Sekundärliteratur erfolgen. Sollte ein im Sinne des nationalen Verfassungsrechts formulierter legitimer Grund für die Einschränkung nachgewiesen werden, wäre im dritten Schritt zu prüfen, ob es möglich ist, die im Gesetzestext erwähnte Einschränkungen als angemessen (III) einzuordnen. D. h. es ist zu beurteilen, ob eine mögliche Einschränkung in einem nachvollziehbaren und sinnvollen Zusammenhang mit dem unter (II) formulierten Ziel der Einschränkung steht (Sweet und Mathews 2008, S. 76) und ob die dabei formulierte konkrete Regel tatsächlich zur Realisierung dieses Ziels beitragen kann (Barak 2012, S. 303 ff.). Sollte dies mit ja beantwortet werden können, ist abschließend eine Entscheidung über die Notwendigkeit (IV) der Einschränkung abzugeben. Ähnlich wie auf der internationalen Ebene wäre hier zu zeigen, dass die im Gesetzestext formulierte, einschränkende Regel – im Vergleich zu anderen Einschränkungen – die am wenigsten restriktive ist (Alexy 2002; Barak 2012). Sind alle diese Bedingungen erfüllt, dann kann eine in der Gesetzgebung formulierte Einschränkung auch auf der nationalen Ebene als *gerechtfertigt einschränkende Regulierung* eingeordnet werden. Sollten allerdings bei einer dieser vier Bedingungen Zweifel bestehen, dann ist eine Gesetzgebung als *ungerechtfertigt einschränkende Regulierung* anzusehen.

## Eine Plausibilitätsprobe mit sechs Fallstudien

Vor dem Hintergrund unseres konzeptionellen Vorschlages zur präziseren Einordnung gesetzlicher Maßnahmen zur Auslandsfinanzierung von ZOs diskutieren wir im folgenden Teil des Artikels den konkreten Charakter solcher Maßnahmen in Deutschland, Österreich, der Türkei, Ungarn, Uruguay und Venezuela. Damit möchten wir den vergleichend-analytischen Mehrwert unserer Heuristik besser verdeutlichen. Methodisch verstehen sich diese sechs kurzen Fallstudien als eine Plausibilitätsprüfung (George und Bennett 2005; Levy 2008), „to give the reader a ‚feel‘ for a theoretical argument by providing a concrete example of its application, or to demonstrate the empirical relevance of a theoretical proposition by identifying at least one relevant case“ (Levy 2008, S. 6–7).

Bei der Auswahl dieser sechs Fälle habe wir uns von zwei zentralen Kriterien leiten lassen. Erstens sollten alle Länder den ICCPR (UN General Assembly 1966) ratifiziert haben und damit im Prinzip dem Gültigkeitsbereich der internationalen Menschenrechte unterliegen. Zweitens sollten in allen zu diskutierenden Fällen spätestens seit Ende der 1990er-Jahre das Thema der Auslandsfinanzierung von Zivilgesellschaft als politisches Problem aufgetreten sein. D. h. es sollte mindestens einen politischen Diskurs über die Notwendigkeit einer verbesserten Regulierung in diesem Bereich gegeben haben.

### Deutschland

Mitte der 2010er-Jahre wurde in der Bundesrepublik debattiert, ob es notwendig sei, für sogenannte Moscheevereine, die Möglichkeit einer Auslandsfinanzierung zu begrenzen. Beflügelt durch die aufstrebende Alternative für Deutschland (AfD) und teilweise unterstützt von Vertretern der Christlich-Sozialen Union (CSU), sprach sich eine Reihe von Politikern für ein zukünftiges Verbot der Auslandsfinanzierung für diesen Teil der Zivilgesellschaft aus (z. B. Tagesspiegel 2016; Welt 2016). Trotz einer intensiv geführten öffentlichen Debatte stellte sich schnell heraus, dass es in Deutschland nicht nötig ist, zusätzliche gesetzliche Maßnahmen für diesen Bereich zu formulieren. Obwohl im Fall der Bundesrepublik das Problem der Auslandsfinanzierung als politisches Problem nur für eine sehr kleine und spezifische Gruppe von ZOs diskutiert wurde, bietet eine zusammenfassende Erläuterung der Gründe, warum es schließlich zu keinen neuen staatlichen Maßnahmen kam, einen anschaulichen Anwendungsfall für die von uns entwickelte Heuristik.

Weder existieren im deutschen Vereins- noch im Stiftungsrecht grundsätzliche Beschränkungen einer ausländischen Finanzierung. Eine einer Verhältnismäßigkeitsprüfung nahe kommende kurze Studie des wissenschaftlichen Dienstes im Deutschen Bundestag kommt daher zu dem Schluss, dass sowohl die generelle Beschränkung der Auslandsfinanzierung als auch eine Offenlegungspflicht von ausländischer Finanzierung durch ZOs nach deutschem Recht verfassungswidrig wäre (Deutscher Bundestag Wissenschaftliche Dienste 2017, S. 4). Das gilt selbstverständlich ebenfalls für Moscheevereine. Hauptgrund dafür ist Artikel 9 Absatz 1 im Grundgesetz, welcher die Vereinsgründung als Grundrecht mit Verfassungsrang festschreibt. Zur Aufrechterhaltung von Vereinstätigkeiten sind zwangsläufig finanzielle Mittel nötig, unabhängig davon ob inländischer oder ausländischer Herkunft (Deutscher Bundestag Wissenschaftliche Dienste 2017, S. 5; Muckel und Hentzschel 2018, S. 26). Eine Einschränkung dieses Grundrechts ist daher nur mit Blick auf den Schutz einer anderen, gleichrangigen Verfassungsnorm angemessen. Auf Grundlage der bunderepublikanischen Verfassungsrechtstradition lässt sich allerdings nur schwer ein Grund finden, der legitim, angemessen oder notwendig wäre, die Auslandsfinanzierung zivilgesellschaftlicher Organisationen einzuschränken (Deutscher Bundestag Wissenschaftliche Dienste 2017, S. 5–6; Muckel und Hentzschel 2018, S. 26). Darüber hinaus würde eine Einschränkung finanzieller Zuflüsse aus anderen EU-Mitgliedstaaten zusätzlich den EU-Regularien zur Antidiskriminierung und zum freien Kapitalverkehr zuwiderlaufen (Deutscher Bundestag Wissenschaftliche Dienste 2017, S. 5).

Damit kann Deutschland als ein Fall eingeordnet werden, der exemplarisch für eine Klasse von Ländern steht, in denen die Auslandsfinanzierung von ZOs zwar als politisches Problem thematisiert wurde, dies aber aufgrund der Anerkennung der Vereinigungsfreiheit als Rechtsnorm auf nationaler, wie auch auf internationaler Ebene zu keiner zusätzlichen gesetzlichen Maßnahme geführt hat.

### Österreich

Auch im österreichischen Vereins- und Stiftungsrecht existieren keine spezifischen Beschränkungen zur Auslandsfinanzierung. Verbunden mit dem Aufstieg der rechts-populistischen Freiheitlichen Partei Österreichs (FPÖ), die seit 2005 einen aggressiven islamophoben Wahlkampf führte (Hafez 2015), hat sich zu Beginn der 2010er-Jahre allerdings eine Diskussion über die mögliche Einschränkung der Auslandsfinanzierung von islamischen Religionsgesellschaften – die in Österreich als Körperschaften des öffentlichen Rechts anerkannt sind – entspannt. Im Gegensatz zum deutschen Nachbarn hat dies in der Alpenrepublik im Februar 2015 zur Verabschiedung einer Novelle des bereits zu Beginn des 20. Jahrhunderts unter der Österreichisch-Ungarischen Monarchie entstandenen Islamgesetzes geführt. Artikel 6 Absatz 2 dieses Gesetzes schreibt seitdem vor, dass Einnahmen islamischer Religionsgesellschaften aus nationalen Quellen stammen müssen (Republik Österreich 2015, S. 3). Damit wurde die ausländische Finanzierung einer sehr spezifischen Gruppe von Organisationen in der österreichischen Zivilgesellschaft durch ein Verbot eingeschränkt. Zwar wurde die faktische Finanzierung aus ausländischen Quellen nicht wirklich unterbrochen,^8^ das österreichische Islamgesetz stellt gleichwohl einen interessanten Fall einer staatlichen Einschränkung dar, die zudem durch ein Urteil des österreichischen Verfassungsgerichtshofes als teilweise gerechtfertigt anerkannt ist (Möchel und Schreiber 2019).

Im Juni 2018 leitete die österreichische Regierung zum ersten Mal rechtliche Schritte auf der Grundlage des neuen Islamgesetzes ein und drohte unter anderem mit der Ausweisung Dutzender Imame. Diese Imame wurden, wie die österreichische Regierung ausführte, direkt vom türkischen Staat bezahlt (Löwenstein 2018), was nach österreichischem Recht, wie oben beschrieben, rechtswidrig sei. Später im selben Jahr reichten zwei dieser Imame eine Klage beim österreichischen Verfassungsgerichtshof ein. Im März 2019 veröffentlichte der Gerichtshof sein Urteil, in dem er die Ausweisung jener Imame bestätigte, die von einer für religiöse Angelegenheiten zuständigen türkischen Regierungsorganisation bezahlt wurden (Möchel und Schreiber 2019). Der Verfassungsgerichtshof begründete sein Urteil mit einer ersten Interpretation der Reichweite von Artikel 6 Absatz 2 des österreichischen Islamgesetzes. Dem Gerichtshof zufolge bezieht sich dieser spezielle Absatz darauf, den Einfluss ausländischer Staaten auf religiöse Gesellschaften in Österreich zu unterbinden. Auch wenn damit das Grundrecht der islamischen Religionsgesellschaften, ihren Bestand durch die freie Wahl von finanziellen Mitteln abzusichern, eingeschränkt wird, erscheint diese spezifische Einschränkung sowohl legitim als auch angemessen. Auf Basis der österreichischen Verfassung muss die Unabhängigkeit aller religiöser Gesellschaften von jeglicher staatlichen Einflussnahme gewahrt sein. Das gilt natürlich auch für andere als den österreichischen Staat.^9^ Abgesehen von diesem Urteil des österreichischen Verfassungsgerichtshofes, welches ausschließlich das Verbot der Finanzierung durch ausländische Regierungen als verfassungskonforme Einschränkung bestätigt, scheint unter österreichischen Verfassungsexperten Einigkeit darüber zu bestehen, dass ein grundsätzliches Verbot der Auslandsfinanzierung für islamische Religionsgemeinschaften den verfassungsrechtlichen Grundsätzen der Gleichheit und Parität widerspräche (so z. B. Sterkl 2014; Dautović und Hafez 2019, S. 18).

Ein gleichlautender Einwand kann – ähnlich wie oben im Fall von Deutschland dargestellt – sowohl aus europäischer als auch aus Sicht der internationalen Menschenrechte formuliert werden. Zudem ist im relevanten Artikel 6 Absatz 2 des Islamgesetz kein Verweis auf ein der in Artikel 22 ICCPR aufgeführten legitimen Ziele zu finden. Aus Sicht der internationalen Menschenrechte und der von uns vorgeschlagenen Heuristik folgend, muss das Verbot von Auslandsfinanzierung im österreichischen Islamgesetz daher als *ungerechtfertigt einschränkende Regulierung* eingeordnet werden.

Zusammenfassend ist Österreich damit als ein Fall zu charakterisieren, für den aus einer nationalen verfassungsrechtlichen Sicht eine zweigeteilte Einordnung vorgenommen werden muss, wobei wichtig ist anzumerken, dass sich die hier behandelten Einschränkungen ausschließlich auf einen spezifischen Teil der österreichischen Zivilgesellschaft beziehen. Während das Verbot der Finanzierung von islamischen Religionsgesellschaften durch andere Staaten als *gerechtfertigt einschränkende Regulierung* einzuordnen ist, kann ein Verbot für nichtstaatliche ausländische Akteure als *ungerechtfertigt einschränkende Regulierung* angesehen werden. Aus einer internationalen menschenrechtlichen Perspektive hingegen ist das Verbot der Auslandsfinanzierung eindeutig als *ungerechtfertigt einschränkende Regulierung* zu klassifizieren.

### Türkei

Nachdem der Türkei Ende der 1990er-Jahre in Aussicht gestellt wurde, möglicherweise in die EU aufgenommen werden zu können, kam es bis Mitte der 2000er-Jahre zu einer umfassenden Reform der türkischen Gesetzgebung. Nicht nur wurden alle einschlägigen menschenrechtlichen Konventionen ratifiziert, sondern es wurden gleichfalls weite Teile der türkischen Gesetzgebung an europäische Anforderungen angepasst (Özçetin und Özer 2015). In diesem Kontext entstand 2004 auch ein neues Vereinsgesetz (Gesetz Nr. 5253), dem 2008 eine Novelle des Stiftungsgesetzes (Gesetz Nr. 5737) folgte (Akbaş 2014). In beiden Gesetzen werden gleichlautende Regeln zur Auslandsfinanzierung formuliert. Obwohl spätestens mit der Ausrufung des landesweiten Ausnahmezustandes nach dem gescheiterten Militärputsch vom 20. Juli 2016 in der Türkei massiv in die Vereinigungsfreiheit und andere Grundrechte eingriffen wurde, verwenden wir die beiden in den 2000er-Jahren entstandenen Gesetze, um sie exemplarisch mit Hilfe unserer Heuristik zu analysieren und anschließend einzuordnen.

Aus Platzgründen diskutieren wir im Folgenden allerdings ausschließlich das Vereinsgesetz. Obwohl weite Teile des Gesetzestextes ausschließlich ermöglichende Regulierungen enthalten, werden in Bezug auf die Auslandsfinanzierung in Artikel 21 zwei einschränkende Regeln formuliert. Zunächst heißt es dort, dass der Erhalt ausländischer Mittel bei den örtlichen Behörden anzuzeigen ist (Republic of Turkey 2004, S. 10). Zudem wird festgelegt, dass Auslandsfinanzierung nur über ein Bankkonto entgegengenommen werden darf (Republic of Turkey 2004, S. 10). Aus Sicht der internationalen Menschenrechte stellen beide Maßnahmen eine *gerechtfertigt einschränkende Regulierung* dar, weil sowohl die Bedingung der Rechtmäßigkeit (i), des legitimen Ziels (ii), als auch der Notwendigkeit (iii) erfüllt sind. Nicht nur liegen die Einschränkungen als Teil eines Gesetzes vor, sie fallen ebenfalls unter die in der Präambel genannten Ziele. Artikel 1 definiert als Zweck des Gesetzes die Regulierung der Aktivitäten, der Pflichten und der Rechnungsführung von Vereinen. Interpretiert man diesen Zweck als staatliches Bestreben Transparenz herzustellen, um beispielsweise Betrug, Korruption, Geldwäsche oder der Finanzierung von Terrorismus vorzubeugen, so fällt dies nach allgemeiner Auffassung unter die legitimen Ziele des ICCPR (Bílková et al. 2019, S. 26–28). Zudem erscheint auch die Pflicht zur Anzeige des Zahlungseingangs und die Anforderung ausländische Finanzmittel ausschließlich über das Bankensystem zu beziehen zu den am wenigsten restriktiven Maßnahmen zu gehören, um das oben genannten Ziel des Gesetzes zu erreichen.

Ein Blick auf die nationale verfassungsrechtliche Ebene ergibt ein ganz ähnliches Bild, da auch hier argumentiert werden kann, dass alle vier von uns diskutierten Bedingungen (Rechtmäßigkeit, Legitimität, Angemessenheit und Notwendigkeit) erfüllt sind. Zunächst ist erneut festzustellen, dass es sich um rechtmäßige Einschränkungen handelt, da sie in Form eines Gesetzes vorliegen. Die türkische Verfassung schreibt die Vereinigungsfreiheit in Artikel 33 fest (Republic of Turkey 1982). Allgemeine Einschränkungen dafür sind in Artikel 13 definiert und umfassen z. B. staatliche Integrität, nationale Souveränität und Sicherheit sowie öffentliche Ordnung, Moral und Gesundheit (Akbaş 2014). Alle in Artikel 1 des Gesetzes genannten Gründe können damit als legitime Ziele anerkannt werden, um den Erhalt finanzieller Zuwendungen aus dem Ausland durch die definierten Regeln einzuschränken. Sie beziehen sich auf das staatliche Ziel den zivilgesellschaftlichen Sektor zu regulieren, mit einem besonderen Blick auf die Rechnungsprüfung. Letzteres erscheint als angemessenes Mittel zur Herstellung von Transparenz, um damit Betrug und Korruption bekämpfen oder Geldwäsche und Terrorismusfinanzierung verhindern zu können. Die Pflicht zur Anzeige der Zahlungen aber auch das Erfordernis, alle ausländischen Zahlungen über ein Bankkonto zu leiten, werden einer Einschätzung der Venedig-Kommission zufolge dafür als angemessene staatliche Mittel betrachtet (Bílková et al. 2019, S. 18). Nicht zuletzt kann argumentiert werden, dass beide Maßnahmen, die am wenigsten restriktiven sind. Damit ist auch das Kriterium der Notwendigkeit in einer demokratischen Ordnung erfüllt. Die im türkischen Vereinsgesetz formulierten Einschränkungen sind daher im Rahmen der von uns vorgeschlagenen Heuristik auch im Kontext des nationalen Verfassungsrechts als *gerechtfertigt einschränkende Regulierung *einzuordnen.

### Ungarn

Nachdem die rechtskonservative Fidesz-Partei 2010 mit einem erdrutschartigen Sieg die ungarischen Parlamentswahlen gewonnen hatten, begannen der daraufhin erneut zum Ministerpräsidenten gewählte Viktor Orbán und seine Gefolgsleute, das existierende politische System Ungarns zu ihren Gunsten umzubauen. Als Teil davon wurde ab etwa Mitte der 2010er-Jahre versucht, dem besonders regierungskritischen Teil der Zivilgesellschaft – ZOs die in der Mehrheit durch Geber aus dem Ausland unterstützt wurden – die Existenzgrundlagen zu entziehen (Móra 2018). Ein im Juni 2017 vom ungarischen Parlament verabschiedetes Gesetz, stellte den vorläufigen Höhepunkt dieser Bemühungen dar. Es schreibt eine Reihe von Einschränkungen der ausländischen Finanzierung von Vereinen und Stiftung fest – beide Organisationsformen sind in Ungarn als Rechtssubjekte anerkannt (Szalai und Svensson 2017). Dazu gehört insbesondere die Pflicht zur Offenlegung ausländischer Finanzierung gegenüber den Behörde, deren öffentliche Benennung auf der Webseite und in Publikationen der Organisation, sowie eine zusätzliche Registrierung im Vereinsregister, sobald eine Organisation mehr als 7,2 Mio. Forint (zum damaligen Zeitpunkt ca. 24.000 €) pro Jahr aus dem Ausland erhalten hat (Móra 2018; Bílková et al. 2019, S. 16).

Das Gesetz formuliert in seiner Präambel, dass es durch die Erhöhung von Transparenz innerhalb der Zivilgesellschaft, besser möglich sei, Geldwäsche zu unterbinden und Terrorismus vorzubeugen. Beides sind Gründe, die in Artikel 22 des ICCPR als legitime Ziele einer Beschränkung der Vereinigungsfreiheit aufgeführt werden (z. B. Bílková et al. 2019, S. 19, 26–27; Bárd 2020). In der ungarischen Verfassung wird die Vereinigungsfreiheit in Artikel 63 garantiert, ohne dass dort auf Bedingungen einer möglichen Einschränkung hingewiesen wird. Allerdings sieht Artikel 7 Absatz 1 der Verfassung vor, das innerstaatliches Recht mit dem Völkerrecht in Einklang zu bringen ist. Mit Hilfe dieser Harmonisierungsklausel, können im ungarischen Fall die in Artikel 22 des ICCPR benannten legitimen Ziele ebenfalls auf der nationalen Ebene zur weiteren Einordnung herangezogen werden. Gleichzeitig bietet diese verfassungsrechtliche Eigenschaft die Möglichkeit alle weiteren Schritte für die internationale und nationale Ebene gemeinsam zu diskutieren.

Während die im ungarischen Fall formulierten Einschränkungen unter Verwendung legitimer Gründe begründet werden, scheint das Kriterium der Notwendigkeit dafür nicht erfüllbar zu sein. Erstens geht die Einführung neuer, zielgerichteter Transparenzvorschriften weit über die bereits bestehenden staatlichen Prüfungsanforderungen hinaus, so wie sie in anderen ungarischen Gesetzen formuliert sind. Im Zweifel können die Behörden daher bereits auf Informationen zur Auslandsfinanzierung zugreifen (Amnesty International 2017). Zweitens kann eine ZO, die gegen das Gesetz verstößt, auch mit ihrer Auflösung bestraft werden. Dies ist bei weitem nicht die am wenigsten restriktive Maßnahme, die ein Staat zur Verfügung hätte. Gerade die Auflösung einer Organisation gilt unter Experten zudem als ausgesprochen restriktiv (European Center for Not-for-Profit Law 2017). Drittens sind diese Maßnahmen, wie der EU-Gerichtshof kürzlich festgestellt hat, nicht gerechtfertigt, um das öffentliche Interesse Ungarns zu schützen. Einerseits ist die Annahme des Gesetzgebers, dass alle ausländischen Finanzmittel eine Bedrohung des öffentlichen Interesses darstellen, diskriminierender Natur, und andererseits scheint die Höhe der im Gesetz festgelegten finanziellen Schwellenwerte nicht notwendig zu sein, um Ungarn vor einer wahrgenommenen Bedrohung zu schützen (*C‑78/18 – Commission v Hungary (Transparence associative)* 2020, S. 18). Damit kann abschließend festgehalten werden, dass die im Jahr 2017 vom ungarischen Parlament verabschiedeten Einschränkungen der ausländischen Finanzierung von ZOs sowohl aus Sicht der internationalen Menschenrechte als auch im Kontext des nationalen Verfassungsrechts als *ungerechtfertigt einschränkende Regulierung* eingeordnet werden müssen.

### Uruguay

Im uruguayischen Rechtssystem wird grundsätzlich zwischen Vereinen und Stiftungen als jeweils eigenständigen Rechtssubjekten unterschieden (Bettoni und Cruz 1999). Nach dem Ende der Militärdiktatur 1985 durchlief das Land einen umfassenden Demokratisierungsprozess, zu dem auch eine Modernisierung seines Rechtssystems gehörte. In diesem Zusammenhang wurde 1999 ein neues Stiftungsgesetz verabschiedet (General Assembly of Uruguay 1999), welches wir im Folgenden als zentrale Quelle einer Einordnung heranziehen.^10^ Das Stiftungsgesetz hat einen allgemeinen Charakter und formuliert insbesondere Regeln für die Gründung, Änderung und Auflösung von Stiftungen. In Bezug auf finanzielle Mittel schreibt es vor, dass bei einer Stiftungsgründung das Startkapital und deren Verwendungszweck benannt werden müssen (Artikel 2 und 3). Das Gesetz unterscheidet jedoch nicht zwischen der in- oder ausländischen Herkunft dieser Mittel (Bettoni und Cruz 1999). Darüber hinaus existieren keine spezifischen Regeln mit Bezug auf eine ausländische Finanzierung. Aufgrund seines allgemeinen Charakters ordnen wir das Gesetz daher sowohl aus Sicht der internationalen Menschenrechte als auch aus einer nationalen verfassungsrechtlichen Perspektive als *ermöglichende Regulierung* ein.

### Venezuela

Im Rechtssystem Venezuelas wird ähnlich wie in allen anderen diskutieren Fällen grundsätzlich zwischen Vereinen und Stiftungen als die zwei zentralen rechtlich anerkannten ZOs unterschieden. In Folge der zunehmend autoritäre Züge annehmenden Präsidentschaft Hugo Chávez, die einherging mit einer massiv ansteigenden Einschränkung von Grundrechten, wurde seit etwa 2010 damit begonnen verschiedenen Teilen des venezolanischen Rechtssystems an die Bedürfnisse der Exekutive anzupassen. In diesem Zusammenhang wurde im Dezember 2010 das Gesetz zur Verteidigung der politischen Souveränität und nationalen Selbstbestimmung durch das Parlament verabschiedet. Darin wurden neue staatliche Maßnahmen in Bezug auf die Auslandsfinanzierung von ZOs formuliert. So beschränkt Artikel 4 die finanziellen Mittel aller ZOs mit einer politischen Orientierung und solcher, die sich für den Schutz von politischen Rechten einsetzen, auf ausschließlich nationale Quellen (La Asamblea Nacional de la República Bolivariana de Venezuela 2010).

Obwohl das Gesetz formal das Kriterium der Legalität erfüllt, ist es aus Sicht der internationalen Menschenrechte nicht in der Lage dem nachgelagerten Kriterium des legitimen Ziels gerecht zu werden. Das z führt diesbezüglich den Schutz der Souveränität und nationalen Selbstbestimmung Venezuelas vor ausländischen Interventionen als Rechtfertigungen auf. Beide Aspekte entsprechen allerdings nicht den in Artikel 22 des ICCPR aufgeführten legitimen Ziele für mögliche Einschränkungen. Aus einer Sicht der internationalen Menschenrechte muss dieses Gesetz daher als eine *ungerechtfertigt einschränkende Regulierung* eingeordnet werden. Was die nationale Ebene anbelangt, so ist in Venezuela die Vereinigungsfreiheit als Grundrecht in der Verfassung festgeschrieben und explizit mit der Ausübung von politischen Tätigkeiten verbunden (Bolivarian Republic of Venezuela 1999 Artikel 67). Zudem genießen in Venezuela ratifizierte internationale Menschenrechtsverträge einen verfassungsrechtliche Vorrang (Bolivarian Republic of Venezuela 1999 Artikel 23). Daher kann gleichlautend zur Einordnung auf Basis der internationalen Menschenrechte auch aus verfassungsrechtlicher Sicht das Gesetz als *ungerechtfertigt einschränkende Regulierung* eingeordnet werden.

Der Vollständigkeit halber halten wir es allerdings für angemessen eine kurze weitergehende Einschätzung dazu abzugeben, wie das Gesetz hätte eingeordnet werden können, wenn Artikel 23 der venezolanischen Verfassung nicht gelten würde. Gemäß Artikel 1 des Gesetzes wird die Einschränkung des Mittelbezugs auf nationale Quellen mit dem Schutz der politischen Souveränität und nationalen Selbstbestimmung begründet, um damit eine mögliche Einmischung von außen in die inneren Angelegenheiten Venezuelas verhindern zu können. Da in Artikel 1 der Verfassung unter anderem Souveränität und Selbstbestimmung als Gründungsprinzipien der Nation verankert sind, könnte das Ziel des Gesetzes damit als legitim eingestuft werden. Allerdings erscheint die im Gesetz formulierte Einschränkung mit einem Blick auf ihre Notwendigkeit als nicht unmittelbar mit dem legitimen Ziel verbunden. Der Zugang zu oder die Verwendung von ausländischen Geldern stellt nicht per se eine Bedrohung für staatliche Souveränität und Selbstbestimmung dar. Es könnte als eine solche Bedrohung bewertet werden, wenn ausländische Geldgeber beispielsweise den Bezug von Finanzmitteln an Bedingungen oder Aktivitäten knüpften, die diese Prinzipien potenziell untergraben würden. Allerdings stellen nicht alle der Aktivitäten, die im Gesetz als politische motiviert aufgeführt werden eine unmittelbare Gefährdung von Souveränität und Selbstbestimmung dar. Maßnahmen, welche beispielsweise Formen der politischen Teilhabe fördern, die wiederum die Selbstbestimmung bestimmter bisher marginalisierter Bevölkerungsgruppen erhöhen, würden im Gegensatz dazu beitragen die diese Gruppen betreffenden Teile der Souveränität und Selbstbestimmung zu verbessern. Kurz gesagt bleibt in diesem Zusammenhang unklar, warum es notwendig ist, zum Schutz der politischen Souveränität und nationalen Selbstbestimmung ein vollständiges Verbot ausländischer Finanzierung zu erlassen. Zusammengefasst müsste daher selbst aus einer Perspektive, die den verfassungsrechtlichen Vorrang internationalen Menschenrechtsverträge ignoriert, die im Gesetz formulierten Einschränkungen als eine *ungerechtfertigt einschränkende Regulierung* eingeordnet werden.

### Eine vergleichende Erörterung der sechs Fallstudien

In Tab. 1 haben wir die wichtigsten Dimensionen unserer Plausibilitätsprobe, d. h. der sechs kurzen Fallstudien zu Deutschland, Österreich, der Türkei, Ungarn, Uruguay und Venezuela zusammengefasst. Zunächst sei uns die Bemerkung gestattet, dass auf Basis unserer Heuristik eine Bewertung der im jeweiligen nationalen Recht kodifizierten restriktiven Qualität der Regulierung von Auslandsfinanzierung für ZOs deutliche differenzierter ausfällt als bisher in der Literatur üblich. Obwohl aus Platzgründen auf eine Analyse weiterer Länderbeispiele verzichtet werden musste, hat sich bereits mit Blick auf die sechs diskutierten Fälle gezeigt, wie vielfältig staatliche Maßnahmen auf einer Skala zwischen ermöglichender Regulierung und Repression sein können und das es als keineswegs ausgemacht gilt, dass jegliche zusätzliche gesetzliche Maßnahme in diesem Bereich automatisch als Restriktion einzuordnen ist (vgl. dazu insbesondere die Spalte zur „Einordnung anhand der internationalen Menschenrechte“ zwischen den sechs Fallstudien und zudem mit der Spalte „Einordnung anhand des nationalen Verfassungsrechts“ in Tab. 1).

**Table 1:** 

Land	Zeitpunkt	Politisches Problem	Neue gesetzliche Maßnahmen	Beschreibung der gesetzlichen Maßnahmen	Geltungsbereich der gesetzlichen Maßnahmen	Einordnung anhand internationaler Menschenrechte	Einordnung anhand des nationalen Verfassungsrechts	Besonderheiten
*Deutschland*	2016	Islamophobie	Nein	Verbot der ausländischen Finanzierung von Moscheevereinen	–	–	–	–
*Österreich*	2015	Islamophobie	Ja	Verbot der ausländischen Finanzierung von islamischen Religionsgesellschaften	Islamische Religionsgesellschaften	Ungerechtfertigt einschränkende Regulierung (Repression)	Gerechtfertigt einschränkende Regulierung in Bezug auf Staaten/ungerechtfertigt einschränkende Regulierung in Bezug auf nichtstaatliche ausländische Akteure (Repression)	Einschränkungen sind aufgrund eines Urteils des nationalen Verfassungsgerichts teilweise gerechtfertigt
*Türkei*	2004	Anpassung an europäische Rechtsnormen aufgrund einer erhofften Mitgliedschaft in der EU	Ja	Melde- und Bankkontogebot	Vereine und Stiftungen	Gerechtfertigt einschränkende Regulierung	Gerechtfertigt einschränkende Regulierung	Gültigkeit nur bis zur Ausrufung des Ausnahmezustands im Juli 2016
*Ungarn*	2017	Eindämmung der Aktivitäten von regierungskritischen Teilen der Zivilgesellschaft	Ja	Zusätzliches Melde- und Offenlegungsgebot, Straf- und Auflösungsandrohung bei Nichteinhaltung	Vereine und Stiftungen	Ungerechtfertigt einschränkende Regulierung (Repression)	Ungerechtfertigt einschränkende Regulierung (Repression)	Einschränkungen sind aufgrund eines Urteils des EU-Gerichtshofes ungerechtfertigt
*Uruguay*	1999	Verrechtlichung des zivilgesellschaftlichen Sektors nach der Demokratisierung in den 1980er-Jahren	Ja	Keine	Stiftungen	Ermöglichende Regulierung	Ermöglichende Regulierung	Es kam bisher nicht zu einer vergleichbaren Novellierung des Vereinsrechts
*Venezuela*	2010	Eindämmung der Aktivitäten von regierungskritischen Teilen der Zivilgesellschaft	Ja	Verbot ausländischer Finanzierung	Politisch aktive Vereine und Stiftungen	Ungerechtfertigt einschränkende Regulierung (Repression)	Ungerechtfertigt einschränkende Regulierung (Repression)	–

Eine weitere ursprünglich wenig naheliegende Beobachtung wird mit einem Blick auf die beiden Spalten „Zeitpunkt“ und „politisches Problem“ deutlich. Beim Thema Auslandsfinanzierung für ZOs handelt es sich aus einer empirischen Sicht um ein Phänomen, welches global gesprochen zeitlich weit gestaffelt aufgetreten ist, zudem auf ganz unterschiedlichen politischen Problemlagen beruht und – wie das Beispiel Deutschland zeigt – nicht zwangsläufig in eine Neugestaltung staatlicher Regulierung münden muss. Gleichzeitig und darauf verweist gerade die Spalte zum „Geltungsbereich der neuen gesetzlichen Maßnahmen“, existiert auch in diesem Bereich ein bereits früher in der Literatur diagnostiziertes Problem für den länderübergreifenden Vergleich von nationalen Rechtsquellen. Für ähnliche politische Probleme und soziale Organisationen existieren länderabhängige Differenzen in Bezug auf regulative Lösungen, die sich auf die jeweils historisch gewachsenen Rechtsordnungen zurückführen lassen. Eine Folge ist, dass die in den Rechtsquellen definierten Rechtssubjekte in ihrer länderübergreifenden Bedeutung voneinander abweichen können und dass abhängig vom betrachteten Land für identische Organisationen, wie zum Beispiel ZOs, eine unterschiedlich spezifische Abdeckung innerhalb der miteinander verglichenen Rechtsquelle existiert (vgl. dazu beispielsweise Bolleyer 2018).

Zudem machen insbesondere die Fälle Deutschland und Uruguay, aber letztlich auch Österreich deutlich, dass die Qualität der Einschränkung von Grundrechten stark invers mit dem demokratischen Konsolidierungsgrad korreliert. Einen Unterschied kann darüber hinaus die verbindliche Einbindung von Staaten in zumindest teilweise gewaltenteilig organisierte supranationale Strukturen machen. Das Beispiel Ungarn verdeutlicht dies anschaulich. In solchen Fällen ist es möglich, dass eine offensichtlich *ungerechtfertigt einschränkende Regulierung* durch ein Gemeinschaftsorgane wie den EU-Gerichtshof als rechtswidrig eingestuft wird.

## Schlussfolgerungen

Die weltweite Zunahme gesetzlicher Maßnahmen zur Beschränkung der ausländischen Finanzierung von ZOs hat eine Debatte ausgelöst, in der die überwältigende Mehrheit der Stimmen argumentiert, dass der historisch einmalige Anstieg staatlicher Eingriffe in diesem Bereich ausschließlich als eine Zunahme an Repression zu interpretieren ist. Damit werden staatliche Maßnahmen in diesem Bereich zu einem wichtigen Bestandteil, der seit knapp 20 Jahren zu beobachtenden Welle von schrumpfenden zivilgesellschaftlichen Räumen. Im Gegensatz dazu hat eine Minderheit von Autor:innen argumentiert, dass Staaten gute Gründe haben können, um den externen Zufluss von Finanzmitteln an die Zivilgesellschaft einzuschränken. Angeregt von dieser Debatte, haben wir in diesem Artikel einen Vorschlag formuliert, um die genaueren Unterschiede des einschränkenden Charakters gesetzlicher Maßnahmen im interregionalen Vergleich besser als bisher sichtbar zu machen. Dabei schlagen wir auf Basis der Interpretation von kodifizierten Rechtsquellen vor, gesetzliche Maßnahmen in Zukunft valider auf einer Skala von *ermöglichender Regulierung*, über *gerechtfertigt einschränkender* bis hin zu *ungerechtfertigt einschränkender Regulierung* einzuordnen. Damit, so glauben wir, steht eine Möglichkeit zur Verfügung, die bisher dominante binäre politikwissenschaftliche Sicht auf einen Teilbereich der staatlichen Regulierung von Zivilgesellschaft zu revidieren. Welches sind abschließend die vielleicht relevantesten Forschungsdesiderate, von denen sich im Anschluss an die in diesem Beitrag dargestellten Überlegungen eine Bearbeitung anbieten würde.

Um einen möglichst umfassenden empirischen Überblick über die Ausprägung des Phänomens zu erhalten, wäre es, erstens, sinnvoll das kodifizierte nationale Recht von möglichst vielen Ländern unter Zuhilfenahme der hier diskutierten Heuristik zu untersuchen und entsprechend einzuordnen. Erst dadurch würde sich ein wirklich repräsentatives und globales Bild in Bezug auf die Einschränkung der Auslandsfinanzierung von ZOs ergeben. Darüber hinaus wäre es mit einer breiteren Datenverfügbarkeit möglich, in einer stärker auf kausale Zusammenhänge fokussierenden vergleichenden Analyse den Ursachen von tatsächlichen Beschränkungen und Repression auf den Grund zu gehen. Vorausgesetzt es wäre in Zukunft möglich, eine größere Anzahl von Fällen anhand des von uns vorgeschlagenen Schemas zu kodieren, ergäben sich damit zudem interessante Fragestellungen hinsichtlich einer getrennten Erklärung für den Beginn von *ermöglichender* auf der einen und g*erechtfertigt einschränkender Regulierung* auf der anderen Seite. Dies könnte wiederum neue Perspektiven auf die Forschung zur Auslandsfinanzierung von Zivilgesellschaft im engeren Sinne eröffnen, aber auch zu einer pointierteren Debatte über schrumpfende zivilgesellschaftliche Räume insgesamt beitragen.

Zweitens ließe sich auf Basis einer breiteren Datenverfügbarkeit zukünftig gezielter der von uns gemachten Einschränkung auf eine rechtsimmanente Analyse begegnen. Gerade für nichtdemokratische politische Systeme wäre es im Anschluss an die Kodierung eines globalen Samples sinnvoll den Einfluss von rechtfertigenden öffentlichen Diskursen im Vorfeld der Änderung gesetzlicher Maßnahmen, die Reaktion der Adressaten von einschränkender Regulierung sowie der politischen Opposition oder, wenn verfügbar, die Rechtsprechung nationaler und internationaler Gerichte mit in eine weiterführende Rechtfertigungsbewertung einzubeziehen. Zudem wäre es für diese politischen Regimetypen ebenfalls nötig, einen genaueren Blick auf rechtswidriges Verhalten durch staatliche Stellen, die Rolle von vage gehaltenen gesetzlichen Bestimmungen und die Reaktion staatlicher Entscheidungsträger auf „unwillkommene“ Rechtsauslegungen durch nationale oder internationale Akteure zu werfen. Damit könnte über unseren in diesem Artikel gemachten Vorschlag hinausgehend, eine mögliche Instrumentalisierung von Recht durch illiberale Akteure empirisch sichtbar gemacht werden.^11^

Drittens wäre zu prüfen, ob es möglich und sinnvoll ist, einige der von uns vorgeschlagenen konzeptionellen Elemente für die vergleichende und länderübergreifende Analyse anderer Grundrechtseinschränkungen heranzuziehen. Insgesamt könnte mit so einer Horizonterweiterung insbesondere ein Beitrag zur allgemeineren politikwissenschaftlichen Repressionsforschung geleistet werden, die bekanntlich Repression überwiegend als gewalttätiges Verhalten bzw. dessen Androhung durch staatliche Akteure operationalisiert. Der von uns vorgeschlagene rechtswissenschaftlich inspirierte und im Kern stärker strukturelle Repressionsbegriff wäre eine interessante Ergänzung zu den bisher dominierenden Forschungsansätzen.

Viertens eröffnet unser konzeptioneller Vorschlag der Einordnung von kodifiziertem nationalem Recht eine Möglichkeit, die vor allem in der politikwissenschaftlichen Subdisziplin der internationalen Beziehungen geführten Debatten über globale Normenkonflikte, durch eine präzisere Operationalisierung anzureichern. Dies ist perspektivisch möglich, weil wir mit unserem konzeptionellen Rahmen über die Analyse diskursiver Begründungen von Politik durch staatliche Akteure hinausgehen und zudem die Verletzung von Rechtsnormen sowohl aus Sicht der internationalen Menschenrechte als auch aus verfassungsrechtlicher Sicht inklusive der dabei existierenden Unterschiede sichtbar machen können.

## Footnotes

1 Zivilgesellschaft lässt sich definieren als „[the] realm of organized social life that is voluntary, self-generating, (largely) self-supporting, autonomous from the state, and bound by a legal order or set of shared rules […] civil society is an intermediary entity, standing between the private sphere and the state“ (Diamond 1994, S. 5). Aus pragmatischen und arbeitsökonomischen Gründen verwenden wir in diesem Artikel zudem eine Definition von zivilgesellschaftlicher Organisation, die sich auf nichtstaatliche und nicht gewinnorientierte formale Organisationen konzentriert, die aufgrund der jeweils länderspezifischen Rechtsordnung Rechtsfähigkeit genießen und welche keine unmittelbaren politischen Interessen in Wahlen verfolgen. D. h. in der Regel und der im deutschsprachigen Raum folgenden Terminologie folgend handelt es dich dabei um Vereine und Stiftungen, die im Englischen überlicherweise als *associations* und *foundations* bezeichnet werden.

2 Eine der wenigen Ausnahmen dazu ist Buyse (2018, S. 980–981).

3 Diese rechtswissenschaftliche Unterscheidung zwischen ermöglichenden und einschränkenden Regulierungen stimmt in weiten Teilen mit Definitionen überein, die in der öffentlichen Verwaltung und der Politikwissenschaft verwendet werden. Hier wird argumentiert, dass gesetzliche Maßnahmen die Funktion haben „[to] achieving public goals using rules or standards of behaviour backed up by sanctions or rewards by the state“ (James 2000, S. 372).

4 Inspiriert ist unser Einordnungsvorschlag von einem in den Rechtswissenschaften als Verhältnismäßigkeitsprüfung (proportionality analysis) bekannt gewordenem Verfahren, welches wir versucht haben, für eine politikwissenschaftliche Analyse fruchtbar zu machen. Die Verhältnismäßigkeitsprüfung ist ein rechtswissenschaftliches System von Abwägungsschlüssen mit dem Ziel, Konflikte zwischen Grundrechten und rechtlichen Prinzipen auf verschiedenen staatsrechtlichen Ebenen zu lösen (vgl. dazu beispielsweise Alexy 2002; Crawford 2011; Barak 2012; Jackson 2014).

5 Damit verstehen wir Rechtfertigung enger als in der Politikwissenschaft üblich, wo sich der Begriff überwiegend auf eine Einordung der Bereitstellung guter Gründe in öffentlichen Debatten zwischen Bürgern oder anderen sozialen Akteuren in Bezug auf politische Sachthemen bezieht (vgl. übersichtsartig Abulof und Kornprobst 2016). Allerdings ist ein rechtswissenschaftlicher Rechtfertigungsbegriff insofern Teil des breiteren politikwissenschaftlichen Verständnisses, als dass es sich dabei um eine kodifizierte Form einer öffentlichen Debatte in einem Bereich des gewaltenteilig ausdifferenzierten politischen Systems handelt. Allerdings ist der von uns gewählte rechtsimmanente Rechtfertigungsbegriff insbesondere innerhalb von nichtdemokratischen politischen Systemen anfällig für exekutive Dominanz und Manipulation. Dies ist eine Einschränkung, die wir insbesondere aufgrund der Tatsache, dass wir uns in diesem Artikel auf eine Operationalisierung mit Hilfe kodifizierter Rechtsquellen konzentrieren, hinnehmen müssen.

6 Diese Liste gilt internationalen Menschenrechtsexperten zufolge als erschöpfend (Kiai 2013, 2016).

7 Als Spezialfall sind solche Verfassungen zu betrachten, die Klauseln über die Wirkung von ratifizierten internationalen Verträgen enthalten.

8 Seit der Einführung des Gesetzes werden die Mittel über private, staatlich registrierte Stiftungen geleitet, für die weiterhin keine Einschränkungen in Bezug auf den Erhalt ausländischer Mittel bestehen. Diese Stiftungen leiten dann wiederum ihre Gelder an islamische Religionsgesellschaften weiter (Felke und Otto 2017).

9 Im Original heißt es wörtlich: „Die Wahrung der Selbständigkeit und Unabhängigkeit gesetzlich anerkannter Kirchen und Religionsgesellschaften vom Staat, aber insbesondere auch von anderen Staaten und deren Einrichtungen, bildet ein im öffentlichen Interesse gelegenes Ziel“ (Möchel und Schreiber 2019).

10 Zu einer vergleichbaren Modernisierung im Vereinsrecht Uruguays ist es bis heute nicht gekommen. Das dafür relevante Gesetz stammt aus dem Jahr 1980. Es fällt daher nicht in den zeitlichen Kontext der Debatten zu schrumpfenden zivilgesellschaftlichen Räumen, in die wir unsere Analyse in diesem Artikel einbetten.

11 Wir sind einem/einer Gutachter:in für die Anregung zu dieser Reflexion zu Dank verpflichtet.

## References

[CR1] Abulof U, Kornprobst M (2016). Introduction: the politics of public justification. Contemporary Politics.

[CR2] Akbaş K (2014). Nonprofit law in Turkey.

[CR3] Alexy R (2002). A theory of constitutional rights.

[CR4] American Bar Association (2015). International and comparative law analysis of the right to and restrictions on foreign funding of non-governmental organizations.

[CR5] Amnesty International (2016). Agents of the people. Four years of “foreign agents” law in Russia: consequences for the society.

[CR6] Amnesty International (2017). Hungary: NGO law a vicious and calculated assault on civil society.

[CR7] Anheier HK (2017). Civil society challenged: towards an enabling policy environment. Economics: The Open-Access, Open-Assessment E-Journal.

[CR8] Anheier HK, Lang M, Koyro C (2018). Civil society in times of change: shrinking, changing and expanding spaces. Social cohesion, global governance and the future of politics.

[CR9] Baldus J (2015). Legal foreign funding restrictions on civil society organizations (CSOs) worldwide.

[CR10] Barak A (2012). Proportionality: constitutional rights and their limitations.

[CR11] Bárd, Petra. 2020. *The Hungarian “lex NGO” before the CJEU: calling an abuse of state power by its name*. Verfassungsblog. https://verfassungsblog.de/the-hungarian-lex-ngo-before-the-cjeu-calling-an-abuse-of-state-power-by-its-name/. Zugegriffen: 15. Sep. 2020.

[CR12] Bethke, Felix, und Jonas Wolff. 2020. *COVID-19 as a threat to civic spaces around the world*. PRIF BLOG. https://blog.prif.org/2020/04/01/covid-19-as-a-threat-to-civic-spaces-around-the-world/. Zugegriffen: 23. Apr. 2020.

[CR13] Bettoni A, Cruz A (1999). El Tercer Sector en Uruguay.

[CR15] Bolivarian Republic of Venezuela (1999). Constitution of Venezuela.

[CR16] Bolleyer N (2018). The state and civil society: regulating interest groups, parties, and public benefit organizations in contemporary democracies.

[CR17] van der Borgh C, Terwindt C (2012). Shrinking operational space of NGOs—a framework of analysis. Development in Practice.

[CR19] Brechenmacher S (2017). Civil society under assault: repression and responses in Russia, Egypt, and Ethiopia.

[CR18] Breen OB (2015). Allies or adversaries? Foundation responses to government policing of cross-border charity. International Journal of Not-for-Profit Law.

[CR20] Brown FZ, Brechenmacher S, Carothers T (2020). How will the coronavirus reshape democracy and governance globally?.

[CR21] Buyse A (2018). Squeezing civic space: restrictions on civil society organizations and the linkages with human rights. The International Journal of Human Rights.

[CR14] Bílková V, Clayton R, Cleveland S, Kuijer M, Kjerulf Thorgeirsdottir H, van Dijk P (2019). Report on Funding of Associations.

[CR23] Carothers T (2006). The backlash against democracy promotion. Foreign Affairs.

[CR24] Carothers T, Brechenmacher S (2014). Closing space: democracy and human rights support under fire.

[CR25] Christensen D, Weinstein JM (2013). Defunding dissent: restrictions on aid to NGOs. Journal of Democracy.

[CR22] Court of Justice of the European Union (2020). C-78/18—Commission v Hungary (Transparence associative).

[CR26] Crawford, Emliy. 2011. Proportionality. In *Max Planck encyclopedia of public international law*. https://opil.ouplaw.com/view/10.1093/law:epil/9780199231690/law-9780199231690-e1459. Zugegriffen: 15. Dez. 2018.

[CR27] Dautović R, Hafez F (2019). Institutionalizing Islam in contemporary Austria: a comparative analysis of the Austrian Islam act of 2015 and Austrian religion laws with special emphasis on the Israelite act of 2012. Oxford Journal of Law and Religion.

[CR28] Deutscher Bundestag Wissenschaftliche Dienste (2017). Ausländische Finanzierung von Vereinen.

[CR29] Diamond L (1994). Rethinking civil society: toward democratic consolidation. Journal of Democracy.

[CR30] Dupuy KE, Prakash A (2018). Do donors reduce bilateral aid to countries with restrictive NGO laws? A panel study, 1993–2012. Nonprofit and Voluntary Sector Quarterly.

[CR31] Dupuy KE, Ron J, Prakash A (2015). Who survived? Ethiopia’s regulatory crackdown on foreign-funded NGOs. Review of International Political Economy.

[CR32] Dupuy KE, Ron J, Prakash A (2016). Hands off my regime! Governments’ restrictions on foreign aid to non-governmental organizations in poor and middle-income countries. World Development.

[CR33] Elkins Z (2010). Diffusion and the constitutionalization of Europe. Comparative Political Studies.

[CR34] European Center for Not-for-Profit Law (2017). Hungarian law on foreign funded NGOs.

[CR36] European Commission for Democracy through Law (Venice Commission), OSCE Office for Democratic Institutions and Human Rights (OSCE/ODIHR) (2018). Hungary: joint opinion on the provisions of the so-called stop Soros draft legislative package which directly affect NGOs (in particular draft article 353A of the criminal code on facilitating illegal migration).

[CR35] European Commission for Democracy through Law, Venice Commission, OSCE Office for Democratic Institutions and Human Rights, OSCE/ODIHR (2014). Joint guidelines on freedom of association: adopted by the Venice Commission at its 101st plenary session (Venice, 12–13 December 2014).

[CR37] Felke, Catharina, und Ferdinand Otto. 2017. Islamgesetz: Warum Österreichs Islamgesetz kein Vorbild ist. In *Zeit Online*. April 4. https://www.zeit.de/gesellschaft/zeitgeschehen/2017-04/islamgesetz-cdu-wahlkampf-oesterreich-deutschland/komplettansicht. Zugegriffen: 30. Juli 2021.

[CR38] General Assembly of Uruguay (1999). Ley N° 17163 Asociaciones Civiles—Fundaciones.

[CR101] George AL, Bennett A (2005). Case Studies and Theory Development in the Social Sciences.

[CR39] Gershman C, Allen M (2006). The assault on democracy assistance. Journal of Democracy.

[CR40] Hafez F (2015). Das IslamG im Kontext islamophober Diskurse. Eine Policy Frame-Analyse zum Politikgestaltungsprozess des IslamG 2015. juridikum.

[CR41] Jackson VC (2014). Constitutional law in an age of proportionality. Yale Law Journal.

[CR42] James O (2000). Regulation inside government: public interest justifications and regulatory failures. Public Administration.

[CR43] Kiai M (2013). Report of the Special Rapporteur on the rights to freedom of peaceful assembly and of association.

[CR44] Kiai M (2016). Analysis on international law, standards and principles—applicable to the foreign contributions regulation act 2010 and foreign contributions regulation rules 2011.

[CR45] La Asamblea Nacional de la República Bolivariana de Venezuela (2010). Ley de Defensa de la Soberanía Política y Autodeterminación Nacional.

[CR46] Levy JS (2008). Case studies: types, designs, and logics of inference. Conflict Management and Peace Science.

[CR47] Löwenstein, Stephan. 2018. Islamgesetz in Österreich: Gehaltsschecks aus der Türkei. FAZ.net. https://www.faz.net/1.5630259 (4 September 2019).

[CR48] Mendelson SE (2015). Why governments target civil society and what can be done in response: a new agenda.

[CR49] Möchel, Kid, and Dominik Schreiber. 2019. Politischer Islam: Staatliche Auslandsfinanzierung von Imamen bleibt verboten. Kurier.at. https://kurier.at/chronik/oesterreich/klare-ansage-ausweisung-von-imamen-rechtlich-unbedenklich/400442569 (4. September 2019).

[CR51] Muckel S, Hentzschel L (2018). Rechtliche Möglichkeiten und Grenzen öffentlicher Finanzierung muslimischen Lebens in Deutschland. Die Finanzierung muslimischer Organisationen in Deutschland.

[CR50] Móra, Veronika. 2018. Democratic backsliding and civil society response in Hungary. In *Civicus.org*. https://www.civicus.org/index.php/re-imagining-democracy/stories-from-the-frontlines/3257-democratic-backsliding-and-civil-society-response-in-hungary. Zugegriffen: 2. Juli 2020.

[CR52] Özçetin B, Özer M (2015). The current policy environment for civil society in Turkey.

[CR54] Poppe AE, Wolff J (2017). The contested spaces of civil society in a plural world: norm contestation in the debate about restrictions on international civil society support. Contemporary Politics.

[CR53] Poppe AE, Wolff J (2016). Ungebetene Gäste – Es gibt kein Recht auf Hilfe für die Zivilgesellschaft in anderen Ländern. Weltsichten.

[CR55] Reddy M (2018). Do good fences make good neighbours? Neighbourhood effects of foreign funding restrictions to NGOs. St Antony’s International Review.

[CR56] Republic of Turkey (1982). Constitution of the Republic of Turkey.

[CR57] Republic of Turkey (2004). Associations law (law no 5253).

[CR58] Republik Österreich (2015). Bundesgesetz über die äußeren Rechtsverhältnisse islamischer Religionsgesellschaften – Islamgesetz 2015.

[CR59] Richter, Thomas. 2018. *Reduced scope for action worldwide for civil society*. GIGA Focus Global, Bd. 5/2018. https://www.giga-hamburg.de/en/publication/reduced-scope-for-action-worldwide-for-civil-society. Zugegriffen: 30. Jan. 2020.

[CR60] Rutzen D (2015). Civil society under assault. Journal of Democracy.

[CR61] Rutzen D (2015). Aid barriers and the rise of philanthropic protectionism. International Journal of Not-for-Profit Law.

[CR62] Sikkink K, Rodríguez-Garavito C, Gomez K (2018). A cautionary note about the frame of peril and crisis in human rights activism. Rising to the populist challenge.

[CR63] Sterkl, Maria. 2014. Experten kritisieren geplantes Islamgesetz. In *DerStandard.at*. https://www.derstandard.at/story/2000006364564/erste-kritik-am-neuen-islamgesetz. Zugegriffen: 5. Sep. 2019.

[CR64] Sweet AS, Mathews J (2008). Proportionality balancing and global constitutionalism. Columbia Journal of Transnational Law.

[CR65] Szalai J, Svensson S (2017). Contested forms of solidarity: an overview of civil society organisations in Hungary and their impact on policy and the social economy.

[CR66] Tagesspiegel. 2016. AfD streitet über Moscheen-Verbot. In *Tagesspiegel.de*. https://www.tagesspiegel.de/politik/hoehenflug-der-partei-gestoppt-afd-streitet-ueber-moscheen-verbot/13383580.html. Zugegriffen: 4. Sep. 2019.

[CR67] UN General Assembly (1966). International Covenant on Civil and Political Rights (ICCPR).

[CR68] Unmüßig B (2016). Civil society under pressure – shrinking – closing – no space.

[CR69] Welt. 2016. Scheuer will ausländische Finanzierung von Moscheen verbieten. In *Welt.de*. https://www.welt.de/newsticker/dpa_nt/infoline_nt/brennpunkte_nt/article154331546/Scheuer-will-auslaendische-Finanzierung-von-Moscheen-verbieten.html. Zugegriffen: 3. Sep. 2019.

[CR70] Wolff J, Poppe AE (2015). From closing space to contested spaces: re-assessing current conflicts over international civil society support.

